# Investigating Diet as the Source ofTetrodotoxin in *Pleurobranchaea maculata*

**DOI:** 10.3390/md12010001

**Published:** 2013-12-27

**Authors:** Serena Khor, Susanna A. Wood, Lauren Salvitti, David I. Taylor, Janet Adamson, Paul McNabb, Stephen Craig Cary

**Affiliations:** 1 Department of Biological Sciences, University of Waikato, Private Bag 3105, Hamilton 3240, New Zealand; E-Mails: sk201@waikato.ac.nz (S.K.); ls161@waikato.ac.nz (L.S.); c.cary@waikato.ac.nz (S.C.C.); 2 Cawthron Institute, Nelson 7042, New Zealand; E-Mails: david.taylor@cawthron.org.nz (D.I.T.); janet.adamson@cawthron.org.nz (J.A.); paul.mcnabb@cawthron.org.nz (P.M.); 3 Department of Chemistry, Otago University, Dunedin 9054, New Zealand

**Keywords:** diet, *Pleurobranchaea maculata*, tetrodotoxin

## Abstract

The origin of tetrodotoxin (TTX) is highly debated; researchers have postulated either an endogenous or exogenous source with the host accumulating TTX symbiotically or via food chain transmission. The aim of this study was to determine whether the grey side-gilled sea slug (*Pleurobranchaea maculata*) could obtain TTX from a dietary source, and to attempt to identify this source through environmental surveys. Eighteen non-toxic *P. maculata* were maintained in aquariums and twelve were fed a TTX-containing diet. Three *P. maculata* were harvested after 1 h, 24 h, 17 days and 39 days and TTX concentrations in their stomach, gonad, mantle and remaining tissue/fluids determined using liquid chromatography-mass spectrometry. Tetrodotoxin was detected in all organs/tissue after 1 h with an average uptake of 32%. This decreased throughout the experiment (21%, 15% and 9%, respectively). Benthic surveys at sites with dense populations of toxic *P. maculata* detected very low or no TTX in other organisms. This study demonstrates that *P. maculata* can accumulate TTX through their diet. However, based on the absence of an identifiable TTX source in the environment, in concert with the extremely high TTX concentrations and short life spans of *P. maculata*, it is unlikely to be the sole TTX source for this species.

## 1. Introduction

Tetrodotoxin (TTX) is a low molecular weight neurotoxin which causes death in humans upon ingestion of only 1–2 mg. It is most famous for causing human fatalities associated with eating puffer fish or fugu [1]. Tetrodotoxin was first isolated from and is named after the puffer fish family, *Tetraodontidae* [2,3]. It has now been identified in a range of phylogenetically diverse marine (e.g., blue ringed octopus, horseshoe crabs, trumpet shells) and terrestrial (e.g., rough-skinned newt, common garter snake, atelopid frogs) organisms [1,4,5]. Among these organisms striking commonalities exist including; TTX and non-TTX-containing populations within species [4,6], significant within-population variability in TTX concentrations [7,8], marked seasonal differences in TTX concentrations within populations [9,10], no correlation between weight and TTX-concentrations [10], differential concentration of TTX among the organs/tissues of TTX-containing organisms [11,12,13] and the ability for adults to invest TTX in their progeny [14,15]. Elucidating the origin of TTX would greatly assist in explaining these observations. However, the ultimate source of TTX in marine and terrestrial ecosystems is still debated with three hypotheses postulated: endogenous [16,17], accumulation through food-chain transmission [18,19,20,21] or through microbial symbionts [22,23,24,25,26,27,28].

Feeding studies have been performed on a wide range of TTX-containing organisms including puffer fish, rough-skinned newts, garter snakes, comb sea-stars, trumpet shells and caddisflies. These have; provided evidence for a dietary source of TTX [18,20,21,29,30], showed differential uptake and conversion of TTX congeners [31,32], explored whether intoxication of non-toxic strains can occur [19,33,34] and investigated the defensive function of TTX [13,15,30,35]. Local variation in puffer fish TTX concentrations [11] in concert with a study by Noguchi, *et al.* [29] in which over 5000 cultured puffer fish were reared in net and land based aquarium with TTX-free diets for 1 to 3 years and became “non-toxic”, indicate a dietary source of TTX in this organism. Additionally, when non-toxic cultured puffer fish were fed with either a TTX-containing diet (toxic puffer fish tissue or liver) [19,31] or TTX-containing bacteria [33], they accumulated the toxin in various parts of their bodies including the skin, liver and ovary. The form of TTX fed to the puffer fish affected accumulation. Puffer fish fed TTX-containing ovary accumulated TTX the fastest and it was detected in all their organs/tissues. Puffer fish fed TTX that was prepared by methanol extraction from toxic organs took longer to accumulate TTX, and no toxins were detected in puffer fish fed crystalline TTX [32].

While the ultimate source of TTX remains a mystery there is little doubt that some species obtain TTX from their diet and use it to increase their fitness. For example, the garter snake (*Thamnophilis sirtalis*) consumes the TTX-containing rough-skinned newt (*Taricha granulosa*) [21,30], the trumpet shell (*Charonia sauliae*) preys on the toxic comb sea-star (*Astropecten polyacanthus*) [20] and caddisfly larvae (*Limnophilus* spp.) feed on the eggs of TTX-containing *T. granulosa* [18]. Although diet is almost certainly the main source of TTX for these and other animals in higher trophic levels, there is far less certainty in organisms such as flat and ribbon worms, frogs and newts. Kim, *et al.* [36] speculated on whether there is a common food source that is widely distributed enough whereby all the different TTX-containing organisms could access it. Under this scenario the most likely producers would be simple single cell organisms such as bacteria and algae, both of which have been implicated in TTX production previously [22,23,26,27].

Tetrodotoxin was first identified in New Zealand in the grey side-gilled sea slug (*Pleurobranchaea maculata*) in 2009 [37]. Populations in Auckland, Whangarei and Tauranga (Upper North Island, New Zealand) were found to contain high concentrations of TTX (up to 1414 mg kg^−1^), while populations in Wellington (Lower North Island, New Zealand) had low concentrations (*ca.* 2.2 mg kg^−1^) and South Island populations had no or very low concentrations of TTX [10,37]. In this study, non-toxic *P. maculata* sourced from the South Island were maintained in aquariums and fed TTX-containing food for up to 39 days to investigate whether; they would survive or be negatively affected by the toxin, and whether they could accumulate TTX, and if they did, how quickly and to where in the organisms this would be transported. One of the features that makes *P. maculata* a very amendable species to study the source of TTX is that it is found in relatively confined, easily accessible, shallow sub-tidal areas and populations can reach extremely high densities (*ca*. 0.8 individual’s m^−2^ [38]). To investigate whether *P. maculata* might obtain TTX from a dietary source in the wild, two extensive benthic surveys were undertaken at sites where dense populations of highly toxic *P. maculata* occurred. 

## 2. Results

The average weight per individual at the start of the experiment was 8.4 g (±6.0 g) and by day 39 this had increased to 19.8 g (±5.4 g). A behavioral measure (time to turn over when placed on back) was used to investigate the possible negative effects of TTX on *P. maculata* during the experiment. This was run once a week throughout the course of the experiment. No significant differences were observed between the TTX-fed and control *P. maculata* during the study (time 0—df = 16, *P* = 0.36, day 7—df = 7, *P* = 2.37, day 14—df = 7, *P* = 2.4, day 21—df = 4, *P* = 2.78, day 28—df = 4, *P* = 2.78 and day 35—df = 4, *P* = 2.78). 

Spiked recovery experiments demonstrated an average enhancement of the TTX peaks during liquid chromatography-mass spectrometry (LC-MS) analysis of 15.8% and all data were adjusted accordingly. Throughout the experiment only TTX was detected; it was not transformed into other variants. The average TTX concentration in ten pieces of TTX-containing agar was 373 mg kg^−1^ (±8 mg kg^−1^). This value was used to estimate the amount of TTX consumed by each individual. All *P. maculata* samples were subjected to lyophilization prior to TTX analysis. A comparison of weights pre- and post-lyophilization demonstrated that approximately 85% of the *P. maculata* samples were liquid. For comparative purposes (as all other research on *P. maculata* to date has presented TTX concentrations in wet weight (ww)) approximate ww are shown in brackets. 

Four egg masses were laid during the experiment. The first (5.39 g) was laid on day one and had a TTX concentration of 0.9 mg kg^−1^ freeze dried weight (FDW; *ca*. 0.14 mg kg^−1^ ww). The individual *P. maculata* that laid this egg mass was harvested on the same day and had a total TTX concentration of 22 mg kg^−1^ FDW (*ca*. 3.3 mg kg^−1^ ww). The remaining three egg masses were laid by one individual on days one, nine and twenty-five (12.46 g, 3.82 g and 7.91 g, respectively). The TTX concentrations in these egg masses progressively increased during the experiment with TTX concentrations of 11.6 mg kg^−1^ FDW (*ca*. 1.7 mg kg^−1^ ww), 129 mg kg^−1^ FDW (*ca*. 19.4 mg kg^−1^ ww) and 292 mg kg^−1^ FDW (*ca*. 43.9 mg kg^−1^ ww), respectively. The individual *P. maculata* was harvested on day thirty-nine and had a total TTX concentration of 361 mg kg^−1^ FDW (*ca*. 54.3 mg kg^−1^ ww).

**Figure 1:**
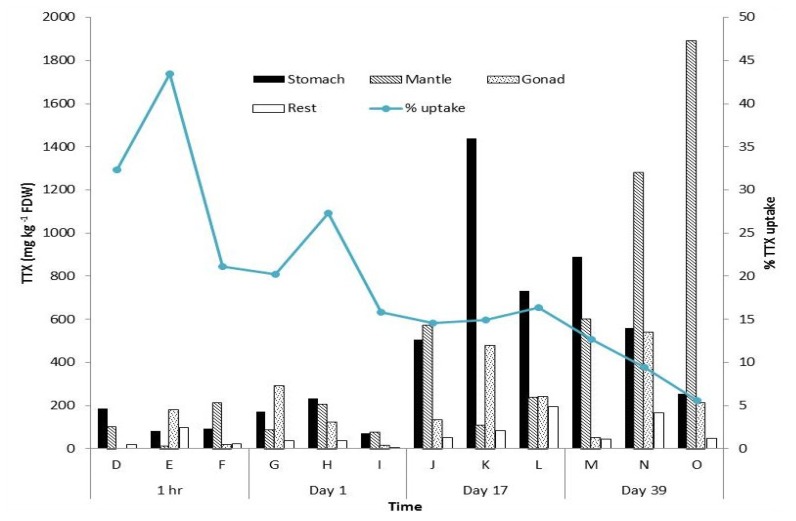
Tetrodotoxin (TTX) concentrations in tissue/organs of *Pleurobranchaea maculata* at various time points after being fed TTX-containing food. Data is presented as amount detected in lyophilized weight (FDW). Each letter represents a single individual.

No TTX was detected in any of the organs/tissues from the three controls harvested prior to the experiment or in the three controls (fed non-TTX containing food) harvested on day 39. Tetrodotoxin was detected in the three *P. maculata* harvested at 1 h. As expected TTX was detected in the stomach (ave. 120 mg kg^−1^ FDW, *ca.* 18 mg kg^−1^ ww). It was also present in reasonably high concentrations in the mantle (ave. 111 mg kg^−1^ FDW, *ca.* 16.6 mg kg^−1^ ww), gonad (ave. 67 mg kg^−1^ FDW, *ca.* 10.1 mg kg^−1^ ww) and “rest” (ave. 46.0 mg kg^−1^ FDW, *ca.* 6.9 mg kg^−1^ ww; Figure 1). The organ/tissue with the highest TTX concentration varied among individuals harvested at this time point. For example, the highest TTX concentration in individual “D” was in the stomach (185 mg kg^−1^ FDW, *ca.* 27.7 mg kg^−1^ ww), whereas individual “E” had the highest TTX concentration in the gonad (182 mg kg^−1^ FDW, *ca.* 27.3 mg kg^−1^ ww) and individual “F” in the mantle (216 mg kg^−1^ FDW, *ca.* 32.5 mg kg^−1^ ww, Figure 1). After 1 h, the average percentage uptake of TTX was 32% (range 21%–43%). After 1 day, the average percentage uptake had decreased slightly (21% (range 16%–27%)) and a similar pattern in variability in TTX concentrations among organs/tissues was observed (Figure 1). After 17 days, the average percentage uptake was 15% (range 15%–16%). Concentrations in individual organs/tissue had increased markedly. In “J” the highest TTX concentration was the mantle (573 mg kg^−1^ FDW, *ca.* 86 mg kg^−1^ ww) whereas “K” and “L” had the highest TTX concentrations in the stomach (1438 mg kg^−1^ FDW, *ca.* 215 mg kg^−1^ ww, and 733 mg kg^−1^ FDW, *ca.* 110 mg kg^−1^ ww respectively; Figure 1). After 39 days the average percentage uptake decreased to 9% (range 6%–13%). Individual “M” had the highest TTX concentrations in the stomach (888 mg kg^−1^ FDW, ww *ca.* 133 mg kg^−1^) whereas “N” and “O” had the greatest TTX concentrations in the mantle (1282 mg kg^−1^ FDW, *ca.* 192 mg kg^−1^ ww and 1890 mg kg^−1^ FDW, *ca.* 284 mg kg^−1^ ww), respectively.

**Figure 2:**
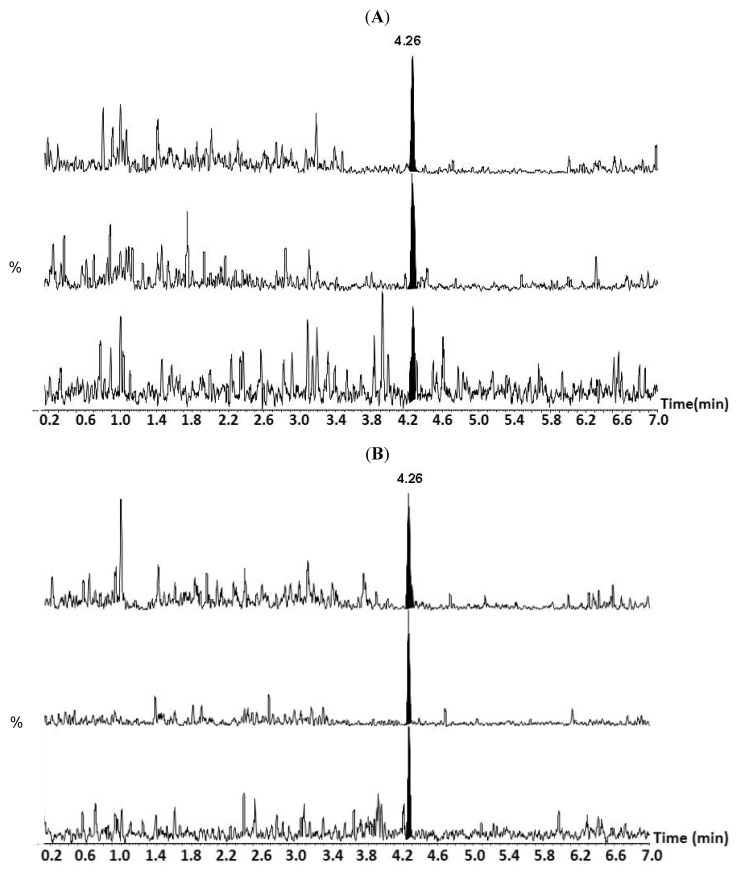
LC-MS/MS chromatograms showing three multiple reaction monitoring (MRM) traces *m*/*z* 320 > 302, 320 > 162 and 320 > 60 from top to bottom (**A**) *Arachnoides zelandiae* with area ratios for each channel of 1.00:0.66:0.16 and (**B**) Authentic TTX at 0.5 ng/mL with area ratios for each channel of 1.00:0.63:0.21. The *x*-axis is time (min) and the *y* axis is LC-MS/MS response normalised to the largest peak in each MRM channel.

Low concentrations of TTX (0.25 mg kg^−1^) were found in sand dollars (*Arachnoides zelandiae*; Table 1; Figure 2) from Narrow Neck Beach. This is the first detection of TTX in this organism. Trace amounts of TTX (<0.2 mg kg^−1^) were found the cats’ eye snail (*Turbo smaragdus*), a crab (*Macrothalamus hirtipes*) and coralline algae (*Corallina officinalis*; Table 1) from Narrow Neck Beach. No TTX or C9 were detected in any of the samples from Illiomama Rock (Table 2).

**Table 1:** Benthic organisms/samples collected for tetrodotoxin (TTX) testing from rocky reef and *Musculista senhousia* (Asian Date Mussel) beds at Narrow Neck Beach and TTX concentrations detected. MS = *M. senhousia* beds.

Scientific Name	Common name/Description	Habitat	TTX Level (mg kg^−1^)
Echinodermata			
*Australostichopus mollis*	Sea cucumber	Reef	0
*Stegnaster inflatus*	Orange elevated cushion star	Reef	0
*Patiriella regularis*	Common cushion star	Reef and MS	0
*Coscinasterias calamaria*	11 Arm star	Reef and MS	0
*Evechinus chloroticus*	Sea urchin (Kina)	Reef	0
*Echinocardium australe*	Heart urchin	MS	0
*Arachnoides zelandiae*	Sand Dollar	Reef	0.25
Mollusca			
*Cominella virgata*	Whelk	Reef	0
*Musculista senhousia*	Asian date mussel	MS	0
*Saccostrea glomerata*	Rock oysters	Reef	0
*Cymatium spengleri*	Whelk	Reef	0
*Penion sulcatus*	Whelk	Reef	0
*Cominella adspersa*	Whelk	Reef and MS	0
*Turbo smaragdus*	Cats eye	Reef	Trace
*Haminoea zealandiae*	Bubble shell slugs	Reef	0
*Cellana radians*	Limpets	Reef	0
*Acanthochitona zelandica*	Chiton	Reef	0
*Cryptoconchus porosus*	Chiton	Reef	0
Polychaeta			
*Perinereis amblyodonta*	Polychaete	Reef	0
Crustacea			
*Plagusia chabrus*	Red rock crab	Reef	0
*Ovalipes catharus*	Paddle Crab	MS	0
*Petrolisthes elongatus*	Porcelain crab	Reef	0
*Chamaesipho columna*	Barnacles	Reef	0
*Macrophthalmus hirtipes*	Crab	MS	Trace
*Pagurus* sp.	Hermit crabs	Reef	0
*Callianassa filholi*	Burrowing shrimp	MS	0
Crustacea			
*Plagusia chabrus*	Red rock crab	Reef	0
*Corallina officinalis*	Coralline turf algae	Reef	Trace
Other			
Sediment from *M. senhousia* beds		MS	0

**Table 2:** Benthic organisms collected for tetrodotoxin (TTX) and C9 testing from Iliomama Rock and TTX concentrations detected. ND not detected.

Scientific or Record Name	Common Name/Description	TTX or C9 level (mg kg^−1^)
Echinodermata		
*Patiriella regularis*	Regular seastar	ND
*Coscinasterias calamaria*	Spiny star	ND
*Echinocardium australe*	Heart urchin	ND
*Arachnoides zelandiae*	Snapper biscuit	ND
*Botryllus schlosseri*	Star ascidian	ND
*Stichopus mollis*	Sea cucumber	ND
Porifera		
*Polymastia* sp.	Common sponge	ND
Unknown orange sponge	Sponge	ND
Mollusca		
*Perna canaliculus*	Greenshell^TM^ mussel	ND
*Bursatella leachii*	Ragged Sea Hare	ND
*Buccinulum lineum*	Lined whelk	ND
Cockle shell	Cockle shell	ND
*Zelithophaga truncata*	Bivalve	ND
*Atrina zelandica*	Horse mussel	ND
*Cleidothaerus albidus*	Bivalve	ND
*Mytilus galloprovincialis*	Blue mussel	ND
*Crassostrea gigas*	Pacific oyster	ND
*Sigapatella novazelandiae*	Circular slipper limpet	ND
Polychaeta		
*Thelepus spectabilis*	Polychaete	ND
Annelida		ND
*Chaetopterus* sp.	Parchment worms	ND
Crustacea		
Crab inside gastropod	Crab	ND
*Pagurus novazelandiae*	New Zealand hermit crab	ND
Algae		
*Codium fragile*	Green alga	ND
*Colpomenia sinuosa*	Brown alga	ND
*Laurencia thyrsifera*	Red alga	ND
*Lithothamnion* sp.	Encrusting red alga	ND
*Lithothamnion* sp.	Encrusting red alga	ND
*Plocamium* sp.	Red alga	ND
*Sargassum sinclairii*	Brown alga	ND
Unknown brown algae	Alga	ND
Unknown red algae	Alga	ND
Other		
*Beania discodermiae*	Bryozoan	ND
Biofilm from gastropod		ND
Biofilm from rock		ND
Biofilm from Scallop shell		ND
Biofilm from shell		ND
*Ciona intestinalis*	Sea squirt	ND
*Cnemidocarpa bicornuta*	Sea squirt	ND
Mussel biofilm		ND
*Pecten novazelandiae* (biofilm)	Scallop	ND
*Plumularia setacea*	Hydroid	ND
Rock with biofilm		ND
Sargassum epifauna		ND
Sediment—top of the tube (1 cm)		ND
Sediment—top of the tube (3 cm)		ND
*Styela plicata*	Tunicate	ND
Unknown ascidian		ND

## 3. Discussion

Using a behavioral response measure (time taken to turn to upright position) we observed no negative influence of the TTX-containing diet on non-toxic *P. maculata*. Studies on puffer fish and newts have demonstrated that TTX-resistance can result from substitution of amino acids in the p-loop regions of skeletal muscle and neuronal Na_v_ channels [39]. Garter snakes and clams have a similar sodium channel mutation-based on TTX/STX resistance induced through ingestion of toxic prey [40,41]. Research on the shore crab *Hemigrapsus sanguineus* and the puffer fish *T. niphobles* have shown that TTX-binding proteins in their hemolymph also assist in resistant to TTX [42,43,44,45]. The mechanism via which *P. maculata* confers its TTX-resistance is unknown, however, results of this study suggest that all populations, regardless of whether they contain TTX or not, have evolved this adaptation. The presence of *P. maculata* populations that are resistant to TTX, yet do not contain it, is intriguing and may indicate that the source of TTX is not available at these locations, or that the ecological advantage provided from containing TTX is no longer necessary.

The results of this study demonstrate that non-toxic *P. maculata*, when fed with TTX-containing food, can rapidly sequester and transport the toxin around their bodies. After 1 hour TTX was detected in all organs/tissues tested. Excluding the stomach, the highest values were detected in the mantle. Wood *et al.* [46] suggested that the main function of TTX in *P. maculata* was as a chemical defense. The rapid transport to the mantle and the high concentration in this tissue on day 17 and 39 support this hypothesis. Four egg masses were laid during the experimental period. It was surprising that TTX was detected in the egg mass laid only 1 day after the first feeding of TTX-containing food. This demonstrates how rapidly *P. maculata* can transport this compound and provides further evidence to support the suggestion that adult *P. maculata* invest TTX into their offspring, presumably to function as a chemical defense [46]. In this study, there was not a pronounced transport of TTX to the gonads as might be expected based on this assumption. The individuals used in this study were juveniles (only two laid egg masses during the experiment) and often during dissection we observed that the gonads were not well developed. 

After 1 hour the *P. maculata* in this study had an average TTX uptake of between 21% and 43%. Tetrodotoxin uptake decreased throughout the experiment (*ca.* 9% by day 39) suggesting that *P. maculata* may have a maximum TTX concentration that they can retain. It is plausible that TTX concentrations may correlate with their level of TTX resistance, for example, Hwang *et al.* [47] showed that puffer fish that were generally non-toxic could become weakly toxic in some habitats. They suggested that these species can accumulate a limited amount of TTX, but because they only have a medium level of resistance to TTX they could not accumulate TTX to the same concentration as toxic puffer fish. Despite the low uptake rates, the concentrations in the *P. maculata* at day 39 (*ca.* 2–300 mg kg^−1^ ww in the highest organs/tissues) were within the ranges of wild populations [10]. Feeding studies on other marine TTX-containing organisms have shown comparable percentage uptakes. Yamamori *et al.* [34] fed non-toxic cultured juvenile puffer fish (*Takifugu niphobles*) crystalline TTX for 30 days, followed by 170 days of a TTX-free diet. Initially the puffer fish accumulated *ca.* 50% of the total TTX administered and this gradually decreased to *ca.* 30% by day 80. Honda *et al.* [19] fed non-toxic cultured puffer fish (*Takifugu rubripes*) TTX-containing diets for 60 days at low doses (less than 3 mouse units (MU) g^−1^) and found that TTX accumulated in the skin and liver but at high doses of TTX (up to 57 MU g^−1^), TTX was sequestered in the liver and ovary. Accumulation rates were age-dependent with puffer fish that were younger than 1 year old having percentage uptakes of 0%–17% whereas 1 year old puffer fish had accumulation rates of more than 30%. The *P. maculata* in the present study were late juvenile-young adult age. This age-group was selected to avoid egg-laying individuals as previous studies have shown that *P. maculata* depurate TTX through their egg masses [46]. Uptake rates in terrestrial organisms appear to be much lower. Caddisflies (*Limnophilus* spp.) fed TTX-containing rough-skinned newt eggs had an uptake of only 0.08%–0.47%, although they were able to retain this for up to 134 days [18]. Likewise, garter snakes sequestered only 0.68%–3.4% of TTX seven days after consuming rough-skinned newts [21]. Williams *et al.* [21] and Gall *et al.* [18] hypothesise that a binding protein may be responsible for TTX sequestration in these species, and that the lower uptake rate may be due to a functional limit on the amount of TTX that can be sequestered.

In this experiment the TTX concentrations measured among *P. maculata* harvested on the same day had less variability than TTX concentrations in wild [10,46]. Variable consumption of a common food source high in TTX could explain this observation, with individuals in the wild that consume more TTX-containing food sequestering higher levels of TTX. Similar observations have been reported for garter snakes with individuals that consumed seven TTX-containing newts having higher TTX concentrations in their liver than those that consumed only one [21]. Despite this, and the demonstration in this study that *P. maculata* can accumulate TTX from their diet, during our environment surveys we were unable to detect any organisms or environmental material (*i.e.*, sediment) that contained TTX concentrations high enough to account for the concentrations of toxin detected in *P. maculata.* Low concentrations were found in the detritivore *A. zelandiae*. The samples of this species were collected from subtidal areas at Narrow Neck Beach where *P. maculata* were abundant, and they may have accumulated TTX from *P. maculata* mucus trails. Previous studies have shown that *P. maculata* mucus contains TTX [48]. The concentrations of TTX in *A. zelandiae* are too low to account for the concentrations in *P. maculata* and this species is unlikely to be a significant food source because of its extremely rigid skeleton. Attempts were made to sample a wide variety of habitats that were covered with diverse biofilms, however, we cannot rule out the possibility that we missed sampling the organism/s that provide the source of TTX to *P. maculata*. Given how abundant *P. maculata* are at these sites, the high concentration of TTX (up to 1414 mg kg^−1^) [10], and their short life span (<1 year), we suggest that a dietary source is unlikely for this species. 

## 4. Experimental Section

### 4.1. Field Sampling and Laboratory Conditions

Eighteen *P. maculata* were collected by divers (11 July 2012) from Tasman Bay (41°3′29″ S, 173°5′28″ E), Nelson, New Zealand. Each was placed in a separate plastic bag containing seawater (300 mL) and transported to the laboratory in an insulated container. *Pleurobranchaea maculata* were maintained in aquariums (19 L), filled with 14 L of filtered seawater (0.22 µm) and aerated using fish tank pumps. Initially due to limited aquariums in some cases two individuals were placed in aquariums (separated by a polystyrene block). After the first sampling, each was maintained in a separate aquarium. Aquarium water was exchanged weekly.

### 4.2. Preparation of Tetrodotoxin-Containing Food

A solution containing TTX was prepared using a homogenous mix of approximately ten *P. maculata* collected in September 2009 from Narrow Neck Beach, Auckland, New Zealand. These *P. maculata* were known to contain high concentrations of TTX [37]. A subsample (20 g) of the homogenate was extracted using 90 mL of Milli-Q water containing 0.1% v/v acetic acid. An aliquot (100 mL) of this was mixed with 5.51 g marine agar powder (Difco™) and 0.5 mL red food colouring (Queen Pillar Box, Australia). The red food colouring was added to assist in visually assessing whether each piece of agar was consumed during the feeding experiment. The mixture was microwaved (1100 W, 1 min 50 s) and poured into petri dishes (*ca.* 15 mL). Once the agar had set, it was sectioned into small pieces (*ca.* 0.24 g) using a sterile scalpel, weighed and placed into 1.7 mL tubes (Axygen). These were stored frozen (−20 °C) and the required amount defrosted immediately prior to each feeding. Ten pieces of agar were kept and extracted to determine the concentration of TTX in the agar. Sub-samples (*ca.* 0.3 g) were extracted using Milli-Q water (*ca.* 2.7 mL) containing 0.1% v/v acetic acid. Each sample was homogenized using an ultrasonic probe (30 s, Heat Systems—Ultrasonics, Inc., Model W—220F). Samples were centrifuged (3000× *g*, 10 min) and an aliquot (100 µL) of the supernatant added to 900 µL of 100% methanol containing 0.1% v/v acetic acid and frozen (−20 °C) for 1 h. Samples were centrifuged (3000× *g*, 10 min) and diluted 1:4 with 100% methanol containing 0.1% v/v acetic acid and analysed for TTX using liquid chromatography-mass spectrometry (LC-MS) as previously described [37]. The detection limit of TTX using the LC-MS method was 0.1 ng mL^−1^.

### 4.3. Spiked Recovery Experiment

To determine if the extracted agar matrix resulted in any suppression or enhancement of the TTX signal during LC-MS analysis a spiked recovery experiment was undertaken. A subsample (1 g) of marine agar (made as per manufacturer’s instructions) was homogenised in 9 mL of Milli-Q water containing 0.1% v/v acetic acid using an ultrasonic probe (30 s). A 2.5 mL aliquot was then added to 22.5 mL of methanol containing 0.1% v/v acetic acid. The solution was frozen (1 h) and centrifuged (3000× *g*, 10 min). An aliquot (5 mL) was added to 45 mL of methanol containing 0.1% v/v acetic acid. Ten aliquots (1 mL) of supernatant were taken and placed in LC-MS vials. Eight were spiked in duplicate with pure TTX (Tocris Bioscience, Cat. No: 1078) to give final concentrations of 1 ng mL^−1^, 2 ng mL^−1^, 10 ng mL^−1^ and 100 ng mL^−1^. The final two vials were not spiked. The samples were analysed for TTX using LC-MS as previously described [37].

### 4.4. Feeding Experiment

Prior to the experiment all *P. maculata* were removed from their aquariums, patted dry using paper towels, weighed and returned immediately to the aquarium. To determine if feeding the TTX-containing food had any negative side-effects, each *P. maculata* was gently placed on its back on the floor of the aquarium and the time taken to turn over was measured prior to the experiment commencing and thereafter once weekly. Individuals were assessed sequentially and in triplicate. 

Three *P. maculata* were harvested as controls prior to commencing the feeding experiment. These were frozen immediately (−20 °C) for later dissection and TTX analysis. Twelve of the remaining *P. maculata* were fed three times a week with TTX-containing food and once a week this was supplemented with *ca.* 0.3 g Greenshell™ mussel (*Perna canaliculus*) sourced from the Marlborough Sounds (South Island, New Zealand). To ensure these mussels were free of TTX, three individuals were tested for TTX as described below. The weight of TTX-containing food ingested was recorded for each individual. Three individuals were harvested at each of the following sampling points after the initial feeding; 1 h, 24 h, 17 days and 39 days. These were frozen immediately (−20 °C) for later dissection and TTX analysis. The remaining three *P. maculata* were fed three times a week with Greenshell™ mussel and harvested at the end of the experiment (39 days). Eggs masses laid during the experiment were collected by gently scraping them off the aquarium wall using a spatula. These were washed (Milli-Q water), weighed and frozen (−20 °C) for later TTX analysis.

### 4.5. Dissection, Tetrodotoxin Extraction and Analysis

*Pleurobranchaea maculata* were partially defrosted and dissected using a sterile scalpel. The gonad, stomach, and a section of the mantle were removed. Emphasis was placed on taking a clean sample rather than on trying to dissect the entire gonad due to difficulties separating the gonad from the stomach. The remaining fluids and tissue were combined, homogenized, and labeled as “rest”. All samples were weighed, frozen (−80 °C) and then lyophilized (FreeZone6, Labconco, MO, USA) and reweighed. 

The freeze dried organ/tissue or egg masses were then ground using a glass pestle. Sub-samples (0.16 g) were extracted with 15.58 mL of Milli-Q water containing 0.1% v/v acetic acid or a *pro rata* volume if the starting mass was greater than 0.16 g. The samples were ultrasonicated for 15 min (T-14, L and R Ultrasonics, Kearny, NJ, USA), centrifuged (3000 × *g*, 10 min) and an aliquot (100 µL) of the supernatant added to 900 µL of 100% methanol containing 0.1% v/v acetic acid and frozen (−20 °C) for at least 1 h. Samples were centrifuged (3000× *g*, 10 min) and diluted 1:4 (100% methanol containing 0.1% v/v acetic acid) and then analysed for TTX using LC-MS as previously described [37].

### 4.6. Statistical Analysis

The total TTX concentration for each *P. maculata* was calculated using the TTX concentration and the proportional weights of the gonad, stomach, mantle, and “rest”. This was then compared with the amount of TTX ingested by each *P. maculata* to estimate percentage uptake. 

The combined averages for the behavioural measure for the control *P. maculata* and TTX-fed *P. maculata* were calculated at each time point. Control and TTX-fed *P. maculata* averages at each time point were then compared using a *t*-test to determine if there was a statistical significance between the two groups using Statistica 8 (StatSoft Inc., Tulsa, OK, USA). 

### 4.7. Environmental Surveys

On 13 July 2010, a survey of benthic organisms on rocky reefs and *Musculista senhousia* (Asian date mussel) beds was undertaken by Scuba divers at Narrow Neck Beach (Auckland, New Zealand). During these surveys, all visible benthic flora and fauna were collected and bagged before freezing; where possible, five specimens of each species were collected (Table 1). The Narrow Neck Beach site had very high densities of *P. maculata* (up to 0.8 m^−2^; [38]) and these contained high levels of TTX [10,37]. In August 2011, a second survey was undertaken by Scuba divers at Illiomama Rock (Auckland, New Zealand) and 51 samples were collected (Table 2). This site also contained high densities of toxic *P. maculata* (up to 0.3 individuals’ m^−2^; [10,38]). The specimens from both surveys commonly consisted of a consortium of species and most were covered with biofilms. The samples were homogenized in their entirety to enable all of these species to be assessed. Sub-samples (2 g) of each were prepared and tested for TTX using LC-MS as described above. All samples from Illiomama Rock were also screened for C9 using the methods described in McNabb [49].

## 5. Conclusions

Non-toxic *P. maculata* were not adversely affected when fed a TTX-containing diet and the TTX was rapidly sequestered and transported within the organisms. Two individuals laid eggs and TTX was also detected in their egg masses demonstrating that *P. maculata* invest TTX in their progeny. The average percentage uptake was similar to the levels reported for other marine organisms and by day 39, TTX concentrations in individuals were similar to those reported from wild populations. Two extensive benthic environmental surveys detected very low (<0.2 mg kg^−1^) or no TTX in samples. This study and previous research on *P. maculata* provide evidence to support and refute; (1) a dietary source of TTX in *P. maculata* (e.g., TTX declines on a TTX-free diet [43], non-toxic *P. maculata* can accumulate TTX from a TTX-containing diet (this study) and no TTX was found during environmental surveys (this study)); (2) endogenously production (e.g., TTX acquisition ceases once egg laying begins [10]); and (3) bacterial production (e.g., shifts in bacterial communities [10] and the inability to isolate TTX-producing bacteria from this species [50]). To date, we can neither exclude nor confirm these possibilities, and the exact origin of TTX in *P. maculata* remains unknown.
